# Influence of Parent–Child Conflict on Psychological Distress among Chinese Adolescents: Moderating Effects of School Connectedness and Neighborhood Disorder

**DOI:** 10.3390/ijerph19159397

**Published:** 2022-07-31

**Authors:** Zhiyou Wang, Ji-Kang Chen

**Affiliations:** Department of Social Work, The Chinese University of Hong Kong, Sha Tin, Hong Kong; jkchen@swk.cuhk.edu.hk

**Keywords:** parent–child conflict, school connectedness, neighborhood disorder, psychological distress

## Abstract

Previous empirical studies have found that not all adolescents showed a high level of psychological distress when facing parent–child conflict, which implies that there could be some additional moderating variables in this pair association. School connectedness and neighborhood disorder have been regarded as possible moderators of this relationship, but empirical evidence is lacking. The participants in this study included 971 students from two middle schools (grades 7–9) and two high schools (grades 10–12) and their parents in the City of Y, Shanxi Province, in mainland China. The PROCESS macro was used to conduct the moderation analysis. The results revealed that both school connectedness and neighborhood disorder significantly moderated the association of parent–child conflict with adolescent psychological distress. These findings highlighted the significance of increasing school connectedness and decreasing neighborhood disorder to alleviate adolescent psychological distress, thereby contributing to related policies and interventions.

## 1. Introduction

Psychological distress refers to a series of physical and psychological symptoms related to normal mood swings in most people, mainly including depression, anxiety, and somatic complaints [1]. It has received global attention because of its detrimental influence on adolescent development such as poor academic performance, school dropout, substance abuse, and even suicide [2]. It is estimated that 13.4% of the global population is suffering from psychological distress [3]. In China, the population with moderate to severe depressive, anxiety, and stress symptoms accounts for 16.5%, 28.8%, and 8.1% of the general population, respectively [4].

Among the factors that induce psychological distress in children, parent–child conflict plays a very prominent role [5]. Especially for teenagers who are in puberty, the biological and cognitive changes lead them to strive for autonomy and individuation, which could heighten conflicts and diminish their closeness with their parents [6]. In addition, Chinese Confucian culture has always emphasized the responsibility of children to obey their parents [7] so children could be more likely to compromise when parent–child conflicts arise, which makes it easier for them to accumulate negative emotions. In addition, several theories and perspectives such as attachment theory and family functioning theory have suggested that adolescents facing conflicts with their parents are highly likely to encounter psychological distress [8]. Children who establish a healthy relationship with their parents could be more likely to form positive expectations for the development of peer relationships. However, parent–child alienation and conflict could make children feel as though it is not worth having a solid relationship [9]. As a result, they could make efforts to resist the surrounding environment causing peer rejection, which in turn increases their risk of psychological distress [10]. Parent–child conflict could also lead to greater psychological distress due to the low level of parental support [11]. However, the results of previous empirical studies showed mixed findings: in facing parent–child conflict, some youth showed high levels of psychological distress, whereas some only showed low-level or even non-significant symptoms [12,13,14]. This indicates that there could be some potential moderating variables in this relationship. The stress-buffering model and integrative model suggest that school connectedness and neighborhood disorder could moderate the link between parent–child conflict and adolescent psychological distress. However, relevant empirical evidence is lacking. Therefore, in order to help improve the mental health of adolescents confronted with parent–child conflict, this study aims to explore the underlying mechanisms between parent–child conflict and psychological distress by testing the moderation effects of school connectedness and neighborhood disorder.

### 1.1. Parent–Child Conflict and Psychological Distress

Parent–child conflict refers to disharmonious or intense interactions during which both the parents and children show negative behaviors and emotions [15] and is recognized as a crucial stressor for adolescents that adversely affects their mental health [12]. According to attachment theory [8], children develop an attachment style based on interactions with their main caregivers. Negative interactions with parents such as parent–child conflict have been considered a risk factor for an insecure attachment style [16], which exacerbates adolescent psychological distress [17]. Theories about family functioning and parenting [18] also support the proposition that children who experience a low-quality relationship with parents are highly likely to suffer from psychological distress.

Although a growing body of investigations has shown a consistent association between parent–child conflict and adolescent psychological distress [13,14], not all adolescents showed a high level of psychological distress when confronted with conflict with their parents [12]. The heterogeneity in the responses to conflict with parents implies that there could be some additional variables moderating the link between parent–child conflict and psychological distress among adolescents. The bio-ecological systems theory offers a guiding framework to explore the potential moderators between parent–child conflict and adolescent psychological distress [19]. The theory argues that human development should be understood in light of different ecological systems, as well as the interactions between the various environmental systems. Therefore, it is reasonable to hypothesize that adolescent psychological distress may not only be directly affected by factors within the family (e.g., parent–child conflict), school (e.g., school connectedness) and neighborhood (e.g., neighborhood disorder) separately but also by the interactions between them.

### 1.2. School Connectedness as Moderator

School connectedness is defined as a positive emotional connection between an individual, the school, and the people at the school, such as peers and teachers, and is regarded as personally perceived external support [20]. Previous studies have shown that students connected closely with school are more likely to enjoy a high level of self-esteem, life satisfaction, and motivation [21,22], whereas students who are less connected with school are highly likely to encounter anxiety, depression, somatization, and other psychological distress [23,24]. In addition, the stress-buffering model also points out that school connectedness can play a protective role against the adverse influence of stressful events on individual psychological distress [25]. Students with a high level of school connectedness are more likely to develop healthy interpersonal relationships with peers, teachers, and other significant people at school and perceived more social support from them [26,27]. Furthermore, a strong connection with school can also increase students’ sense of security and encourage them to take on more meaningful roles and adaptive coping skills [28]. Those sources could help them to attenuate the harmful influence of parent–child conflict. However, there is a lack of empirical studies on the moderating effect of school connectedness on adolescent psychological distress related to parent–child conflict.

### 1.3. Neighborhood Disorder as Moderator

Neighborhood disorder is a sociological construct referring to the physical and social signs of menace and risk in the neighborhood [29]. It has been regarded as a great threat to adolescent development [30,31]. The stress process model asserts that individuals who have daily exposure to a threatening environment are more likely to experience great stress, triggering psychological distress [32]. Empirical studies have also found that adolescents living in a disordered neighborhood characterized by frequent violence, alcohol use, and graffiti will perceive intense stress, increasing their vulnerability to psychological distress [33]. In addition, according to the integrative model [34], the nexus between family socialization processes and children’s developmental outcomes differs depending on specific ecological circumstances such as neighborhood disorder. That is, neighborhood disorder can moderate the effects of parent–child conflict on adolescent psychological distress. The mainstream perspective on neighborhood disorder has further postulated that a disordered neighborhood environment can intensify the detrimental impact of parent–child conflict on adolescent psychological distress [35]. Similarly, relevant empirical evidences are lacking to support these perspectives.

In summary, based on the above statement, we propose the following research hypotheses:

**Hypothesis** **1.** *School connectedness can attenuate the association between parent–child conflict and adolescent psychological distress*.

**Hypothesis** **2.** *Neighborhood disorder can reinforce the association between parent–child conflict and adolescent psychological distress*.

## 2. Materials and Methods

### 2.1. Participants and Procedures

The participants consisted of 971 adolescents in middle or high school (in grades 7–12) and their parents in Y city, in Shanxi Province, China, using multi-stage cluster random sampling. The adolescent sample included 469 boys and 468 girls. Fifty-six students (5.8%) were from single-parent families, and 908(93.5%) came from two-parent families. First, based on the list obtained from the Y government, two counties were randomly selected. Subsequently, a middle school and a senior high school were randomly chosen in each county. Next, one to three classes were chosen randomly from each grade in each selected school. Finally, all the students in every selected class were chosen to take part in this survey. Before data collection, consent forms were handed out to all students and their parents. Research assistants guided the students to finish the questionnaires independently in class. Students took home the part of the questionnaire to be completed by their parents and returned the completed questionnaires to school the next day. Ethical standards were strictly followed throughout the process, and this investigation was approved by the Survey and Behavioral Research Ethics Committee of the first author-affiliated university.

### 2.2. Measures

#### 2.2.1. Parent–Child Conflict

The subscale of parent–child conflict from the parental environment questionnaire (PEQ) was used to assess the level of parent–child conflict [36]. We translated this scale into Chinese using the back-translation method because the scale has not yet been used or validated in the Chinese context. After conducting confirmatory factor analysis (CFA), the items with factor loadings less than 0.4 were deleted so as to increase research validity [37,38]. Each item was measured on a 4-point Likert scale (from 1 = never to 4 = often). All eleven items were summed and higher overall scores reflected more serious parent–child conflict. Cronbach’s α for the scale in this study was 0.900.

#### 2.2.2. School Connectedness

Five items selected from the National Longitudinal Study of Adolescent Health were used to measure adolescents’ perceived school connectedness [39]. Respondents were required to indicate their agreement or disagreement with five questions such as “I feel close to people in my school.” Participants responded on a five-point Likert scale ranging from (1) strongly disagree to (5) strongly agree. The responses were summed with higher scores indicating a higher level of school connectedness. The Chinese version of this scale has also proved good internal consistency in previous studies [40]. In this study, Cronbach’s α was 0.800.

#### 2.2.3. Neighborhood Disorder

The perceived neighborhood disorder scale (PNDS) was applied to measure teenagers’ perceptions of neighborhood disorder [41]. Since the scale has not yet been applied to the Chinese population in previous studies, we translated it into Chinese based on the back-translation method. CFA was carried out and items with factor loadings exceeding 0.40 were required based on the guidelines [37,38]. Five items assessing neighborhood disorder were selected. Participants were asked to respond to statements such as “My neighborhood is very safe” and “I can trust most people in my neighborhood.” Each item was measured on a 4-point Likert scale ranging from (1) strongly agree to (4) strongly disagree. Scores of all items were summed with a higher score implying a higher level of neighborhood disorder. The Cronbach’s α of this scale was 0.821 in this study.

#### 2.2.4. Psychological Distress

Three subscales of depression, anxiety, and somatization were selected from the Brief Symptom Rating Scale to construct the dependent variable of psychological distress in the present study [42]. Both the depression and anxiety subscales included seven items such as “Have suicidal thoughts” and “Feel nervous.” The somatization comprises four items such as “Muscle pain.” Participants responded to each item on a five-point Likert scale (1 = never, 2 = slight, 3 = medium, 4 = severe, 5 = very severe). The responses were summed with higher scores suggesting a higher level of psychological distress. The Chinese version of the scale was found to have good reliability and validity [43]. In this study, the Cronbach’s α for this scale was 0.924.

#### 2.2.5. Covariates

Adolescents’ gender (1 = male; 2 = female) and grade (1–3 representing grades 7–9 in middle school and 4–6 representing grades 10–12 in high school), parent’s gender (1 = male; 2 = female), and single-parent family status (1 = yes; 2 = no) were controlled.

### 2.3. Analytic Strategy

All analyses were conducted in SPSS 23.0. The expectation-maximization (EM) estimation method was used to deal with the missing values; the data used had less than five percent of missing values [44]. Next, descriptive statistics for the main variables were calculated including the means and standard deviations. Pearson correlations were also computed. Then, the PROCESS macro was performed to examine the moderation models [45]. Two separate moderation analyses were conducted to investigate the hypothetical moderation effects of school connectedness and neighborhood disorder on the link between parent–child conflict and psychological distress, controlling for adolescent gender, grade, parent gender, and single-parent family status. Continuous variables were mean-centered, and 95% bias-corrected percentile bootstrap confidence intervals (CI) with 5000 samples were used to assess the significance of the moderation effects. When the CI did not include 0 at the 95% level, the result was significant or non-significant if otherwise. In addition, simple slope tests were conducted to examine the association between parent–child conflict and adolescent psychological distress, with high versus low levels (1SD above and below the mean) of school connectedness and neighborhood disorder. Furthermore, before conducting the moderation analysis, the multicollinearity of the predictors (except for the interaction variables) was examined and the results were in an acceptable range (VIFs < 1.09).

## 3. Results

### 3.1. Descriptive Analyses and Correlations between the Core Variables

Table 1 shows the means, standard deviations (SD), and Pearson correlations for all core variables in the current study. The results show that adolescents’ gender, grade level, and single-parent family status were linked significantly to adolescent psychological distress (*r* = 0.089, *p* < 0.01 for gender; *r* = 0.226, *p* < 0.01 for grade; *r* = −0.066, *p* < 0.05 for single-parent family status). In addition, parent–child conflict was significantly negatively linked with school connectedness (*r* = −0.186, *p* < 0.01) and positively linked with neighborhood disorder (*r* = 0.180, *p* < 0.01) and adolescent psychological distress (*r* = 0.278, *p* < 0.01). School connectedness was negatively associated with neighborhood disorder (*r* = −0.409, *p* < 0.01) and adolescent psychological distress (*r* = −0.428, *p* < 0.01), and neighborhood disorder was positively associated with adolescent psychological distress (*r* = 0.378, *p* < 0.01).

### 3.2. Moderating Effects of School Connectedness and Neighborhood Disorder

**Figure 1:**
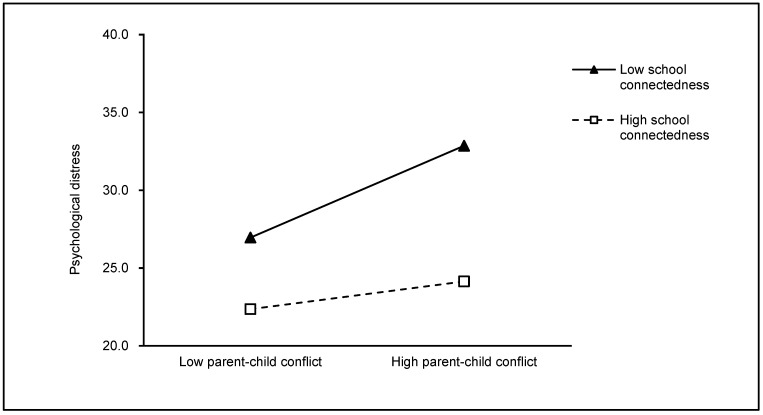
Impact of interaction between parent–child conflict and school connectedness on adolescent psychological distress.

**Figure 2:**
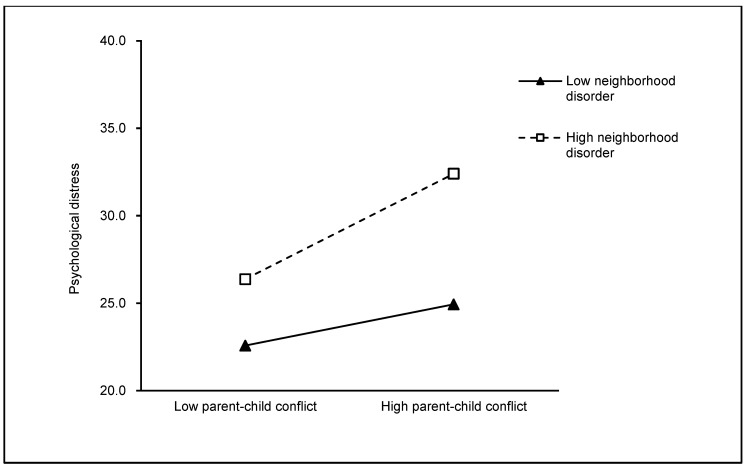
Interaction between parent–child conflict and neighborhood disorder on adolescent psychological distress.

Table 2 presents the results of the moderation analysis using the bootstrapping method, and shows that the two main moderation models were reliable. In model 1, school connectedness was tested as a moderator in the relationship between parent–child conflict and adolescent psychological distress, R^2^= 0.266, F(7, 963) = 49.838, *p* < 0.001. The interaction between parent–child conflict and school connectedness was significant (*b* = −0.041, SE = 0.011, *t* = −3.747, *p* < 0.001, 95%CI = [−0.062, −0.019]), which indicates that school connectedness significantly moderated the link between parent–child conflict and adolescent psychological distress. So, hypothesis 1 is supported in this study. Model 2 tested the moderation effect of neighborhood disorder on the link between parent–child conflict and adolescent psychological distress, R^2^ = 0.234, F(7, 963) = 42.003, *p* < 0.001. The results also show a significant interaction between parent–child conflict and neighborhood disorder (*b* = 0.049, SE= 0.014, *t* = 3.574, *p* < 0.001, 95%CI = [0.022, 0.076]), suggesting that neighborhood disorder significantly moderated the relationship between parent–child conflict and adolescent psychological distress. Thus, hypothesis 2 is also supported. In addition, for descriptive purposes, the predicted adolescent psychological distress was plotted against parent–child conflict separately for low and high levels of school connectedness and neighborhood disorder. As depicted in Figure 1, the results of the simple slope tests suggest a significant positive link between parent–child conflict and adolescent psychological distress among students with high levels of school connectedness (*b* = 0.154, SE = 0.058, *t* = 2.682, *p* < 0.01, 95% CI = [0.041, 0.267]), whereas the association became stronger among students with low levels of school connectedness (*b* = 0.456, SE = 0.055, *t* = 8.140, *p* < 0.001, 95% CI = [0.338, 0.553]). The conditional effects of parent–child conflict on adolescent psychological distress under the significant moderating effect of neighborhood disorder are shown in Figure 2. The results indicate a significant influence of parent–child conflict on adolescent psychological distress among students living in areas with high levels of neighborhood disorder (*b* = 0.458, SE = 0.056, *t* = 8.155, *p* < 0.001, 95% CI = [0.348, 0.568]). However, for the group living in areas with low levels of neighborhood disorder, although this link remained significant, it became weaker (*b* = 0.183, SE = 0.056, *t* = 3.224, *p* < 0.01, 95% CI = [0.071, 0.295]).

## 4. Discussion

In the present study, we explored how parent–child conflict influences adolescent psychological distress in Chinese society by examining the possible moderators of school connectedness and neighborhood disorder. The results found that school connectedness attenuates the negative influence of parent–child conflict on adolescent psychological distress, whereas neighborhood disorder aggravates the influence. These results respond to the divergence in the results of previous studies regarding the relationship between parent–child conflict and psychological distress among adolescents: in the face of high-intensity parent–child conflict, some teenagers showed high levels of psychological distress, whereas other teenagers showed low levels or no significant symptoms [12,13]. The findings imply that there are indeed some variables that moderate the effects of parent–child conflict on adolescent psychological distress such as school connectedness and neighborhood disorder. Specifically, parent–child conflict predicted a high level of psychological distress among youth with a low level of school connectedness, whereas this link became weaker for teenagers with a high level of school connectedness. The finding is congruent with the stress-buffering model, indicating that school connectedness could help adolescents bounce back and recover from parent–child conflict [25]. A possible reasonable explanation comes from Relational Developmental Systems Theory [46], which proposes that if individuals can participate in positive interactions with the environment, they can achieve good development through system changes, although they could also face many adversities and challenges.

Furthermore, the present study also found that neighborhood disorder moderated the influence of parent–child conflict on adolescent psychological distress. This finding implied that the impact of the family environment on children’s mental health would differ depending on other specific ecological circumstances such as neighborhood disorder [34]. Due to the lower security measures and collective efficacy, residents living in disordered neighborhoods tend to have weaker interactions with their neighbors, which is associated with a low level of social support, thereby increasing the risk of adolescent psychological distress [47]. Another potential explanation is that a disordered neighborhood represents a disadvantaged parenting environment, a high probability of corporal punishment, a lack of positive parenting role models, and limited educational opportunities and resources [48]. Therefore, adolescents living in such neighborhoods were highly likely to be exposed to various adverse events such as child abuse and family dysfunction. Those negative experiences could then aggravate the symptoms of psychological distress in adolescents [49].

This study has made several contributions to theory and social work practices. In theory, some perspectives of the theories involved in this study (e.g., bio-ecological system theory, attachment theory, stress-buffer model, and the integrative model) have been examined within the Chinese context. The study extended the current literature by providing empirical evidence for the proposed theoretical model, which specified that both school connectedness and neighborhood disorder moderate the link between parent–child conflict and adolescent psychological distress. The findings also speak to the divergence of the results of previous studies regarding the association between parent–child conflict and adolescent psychological distress. This theoretical model can be tested in other groups and cultural contexts to explore the mechanisms underlying the influence of parent–child conflict on psychological distress.

In practice, the findings of the present study can guide effective intervention programs to prevent and reduce adolescent psychological distress. First, given that parent–child conflict was shown to the risk of psychological distress among adolescents, efforts to develop a positive relationship between parents and children could prevent the emergence of psychological distress during puberty. Disrupted parenting and a lack of family communication have been demonstrated to be the main factors contributing to parent–child conflict [50]. Thus, social workers can help parents to develop effective educational methods and interact with their children in a friendly manner, thereby promoting the parent–child relationship. Consequently, adolescent psychological distress can be alleviated. In addition, our findings demonstrated that the adverse effects of parent–child conflict on adolescent psychological distress could be attenuated by school connectedness. Therefore, intervention programs focusing on enhancing school connectedness could be useful for reducing adolescent psychological distress when faced with parent–child conflict. School personnel should be committed to establishing and developing a healthy school environment characterized by a high level of school support, acceptance, and close connections [51]. A social worker can help students build harmonious relationships with peers and teachers at school so as to mitigate the adverse effects of parent–child conflict on psychological distress [52]. Finally, recognizing the intensifying effects of neighborhood disorder on the nexus between parent–child conflict and adolescent psychological distress will help to identify adolescents who are more vulnerable to psychological distress. Our findings indicated that social work programs should improve the poor conditions of neighborhoods so as to help reduce the negative impact of parent–child conflict on youth. Furthermore, it is important to advocate for related policies dedicated to solving the structural basis of neighborhood disorder so as to build a healthier and safer neighborhood environment, which is very important for the development of children. Interventions aiming at reducing disorder in neighborhoods and increasing social cohesion among residents should be designed and implemented. Promise Neighborhoods in Baltimore is worth learning from. These interventions provide community residents with opportunities to cooperate in improving many aspects of neighborhoods, including housing repairs, public safety, employment, educational interventions, community resources integration, and ultimately improve the well-being of residents [53].

The present study still had some notable limitations. First of all, the cross-sectional data used in this inquiry made it difficult to draw causal conclusions regarding the observed relationships among the variables. Longitudinal investigations should be used to confirm the adverse influence of parent–child conflict on adolescent psychological distress. Second, the current study utilized a sample of middle and senior high school students in the City of Y, Shanxi Province, in mainland China. Therefore, we could not generalize the findings of this study to other cultures and groups. Future research can examine the extent to which the proposed theoretical framework applies to other countries, societies, and populations. Third, the current study only examined school connectedness and neighborhood disorder as moderators, which, respectively, diminish and intensify, the adverse impact of parent-child conflict on adolescent psychological distress. For example, people in Western countries are encouraged to maintain their independence and individuality, whereas Chinese culture places more emphasis on family harmony and connectedness [54]. So, future research is expected to expand the range of variables that could act as mediators and deeply explore the underlying mechanisms of the nexus between parent–child conflict and adolescent psychological distress. Finally, because of the sensitivity of the issue of parent–child conflict, parents could have underreported or overreported real conflicts with their children. Multi-informant data could be collected in future studies to obtain more comprehensive information about parent–child conflict.

## 5. Conclusions

This study found that both school connectedness and neighborhood disorder significantly moderated the association between parent–child conflict and adolescent psychological distress. Specifically, school connectedness attenuates the negative influence of parent–child conflict on adolescent psychological distress, whereas neighborhood disorder aggravates the influence.

## Figures and Tables

**Table 1:** Means, SDs, and correlations between key variables.

Variables	M	SD	1	2	3	4
1.Parent–child conflict	23.684	6.626	_			
2.School connectedness	18.545	3.597	−0.186 **	_		
3.Neighborhood disorder	9.982	2.813	0.180 **	−0.409 **	_	
4.Psychological distress	26.741	9.500	0.278 **	−0.428 **	0.378 **	_

** *p* < 0.01.

**Table 2:** Results of moderation analysis with bootstrapping method.

	Model 1 (School Connectedness as Moderator)						Model 2 (Neighborhood Disorder as Moderator)				
Variables	*b*	SE	*t*	*p*	CI	Variables	*b*	SE	*t*	*p*	CI
Adolescent gender	0.742	0.472	1.573	0.116	[−0.184, 1.667]	Adolescent gender	1.302	0.480	2.716	0.007	[0.361, 2.243]
Grade	0.772	0.142	5.446	<0.001	[0.494, 1.050]	Grade	0.812	0.145	5.590	<0.001	[0.527, 1.100]
Parent gender	−1.420	0.615	−2.310	0.021	[−2.626, −2.214]	Parent gender	−1.120	0.627	−1.785	0.075	[−2.351, 0.111]
Single parent family status	−1.557	0.911	−1.709	0.088	[−3.345, 0.231]	Single parent family status	−1.886	0.929	−2.030	0.042	[−3.709, −0.062]
Parent–child conflict	0.300	0.041	7.400	<0.001	[0.221, 0.380]	Parent–child conflict	0.321	0.041	7.751	<0.001	[0.240, 0.402]
School connectedness	−0.935	0.076	−12.262	<0.001	[−1.082, −0.785]	Neighborhood disorder	0.995	0.099	10.021	<0.001	[0.800, 1.190]
Parent–child conflict × school connectedness	−0.041	0.011	−3.747	<0.001	[−0.062, −0.019]	Parent–child conflict × Neighborhood disorder	0.049	0.014	3.574	<0.001	[0.022, 0.076]
R^2^	0.266					R^2^	0.234				
F	49.838 ***					F	42.003 ***				

Note: SE: Standard error; CI: bootstrapping confidence intervals at 95% level. *** *p* < 0.001.

## Data Availability

Not applicable.

## References

[1] Kessler R.C., Barker P.R., Colpe L.J., Epstein J.F., Gfroerer J.C., Hiripi E., Howes M.J., Normand S.-L.T., Manderscheid R.W., Walters E.E. (2003). Screening for Serious Mental Illness in the General Population. Arch. Gen. Psychiatry.

[2] Copeland W.E., Angold A., Shanahan L., Costello E.J. (2013). Longitudinal Patterns of Anxiety From Childhood to Adulthood: The Great Smoky Mountains Study. J. Am. Acad. Child Adolesc. Psychiatry.

[3] Polanczyk G.V., Salum G.A., Sugaya L.S., Caye A., Rohde L.A. (2015). Annual Research Review: A meta-analysis of the worldwide prevalence of mental disorders in children and adolescents. J. Child Psychol. Psychiatry.

[4] Wang C., Pan R., Wan X., Tan Y., Xu L., Ho C.S., Ho R.C. (2020). Immediate Psychological Responses and Associated Factors during the Initial Stage of the 2019 Coronavirus Disease (COVID-19) Epidemic among the General Population in China. Int. J. Environ. Res. Public Health.

[5] Imrie S., Zadeh S., Wylie K., Golombok S. (2020). Children with Trans Parents: Parent–Child Relationship Quality and Psychological Well-being. Parenting.

[6] Crone E.A., Van Duijvenvoorde A.C.K., Peper J.S. (2016). Annual Research Review: Neural contributions to risk-taking in adolescence —Developmental changes and individual differences. J. Child Psychol. Psychiatry.

[7] Deutsch F.M. (2006). Filial Piety, Patrilineality, and China’s One-Child Policy. J. Fam. Issues.

[8] Mikulincer M., Shaver P.R. (2012). An attachment perspective on psychopathology. World Psychiatry.

[9] Yan J., Feng X., Schoppe-Sullivan S.J. (2018). Longitudinal associations between parent-child relationships in middle childhood and child-perceived loneliness. J. Fam. Psychol..

[10] DeKlyen M., Greenberg M.T., Cassidy J., Shaver P.R. (2016). Attachment and psychopathology in childhood. Handbook of Attachment: Theory, Research, and Clinical Applications.

[11] Cavanaugh A.M., Buehler C. (2015). Adolescent loneliness and social anxiety. J. Soc. Pers. Relatsh..

[12] Branje S.J.T., Hale W.W., Frijns T., Meeus W.H.J. (2010). Longitudinal Associations Between Perceived Parent-Child Relationship Quality and Depressive Symptoms in Adolescence. J. Abnorm. Child Psychol..

[13] Ehrlich K.B., Miller G.E., Chen E. (2015). Harsh parent–child conflict is associated with decreased anti-inflammatory gene expression and increased symptom severity in children with asthma. Dev. Psychopathol..

[14] Samek D.R., Wilson S., McGue M., Iacono W.G. (2016). Genetic and Environmental Influences on Parent-Child Conflict and Child Depression Through Late Adolescence. J. Clin. Child Adolesc. Psychol..

[15] Weaver C.M., Shaw D.S., Crossan J.L., Dishion T.J., Wilson M.N. (2014). Parent–Child Conflict and Early Childhood Adjustment in Two-Parent Low-Income Families: Parallel Developmental Processes. Child Psychiatry Hum. Dev..

[16] Bradford A.B., Burningham K.L., Sandberg J.G., Johnson L.N. (2016). The Association between the Parent-Child Relationship and Symptoms of Anxiety and Depression: The Roles of Attachment and Perceived Spouse Attachment Behaviors. J. Marital Fam. Ther..

[17] Nowakowski-Sims E., Rowe A. (2017). The relationship between childhood adversity, attachment, and internalizing behaviors in a diversion program for child-to-mother violence. Child Abus. Negl..

[18] Conger R.D., Ge X., Elder G.H., Lorenz F.O., Simons R.L. (1994). Economic stress, coercive family process, and developmental problems of adolescents. Child Dev..

[19] Bronfenbrenner U. (1979). The Ecology of Human Development: Experiments by Nature and Design.

[20] Gao Y., Zhang W., Deng Q., Sun C., Gao F., Chen Y. (2020). Shyness and social adjustment in Chinese college students: A moderated mediation of alienation and school connectedness. Curr. Psychol..

[21] Arif S., Khan S., Rauf N.K., Sadia R. (2019). Peer Victimization, School Connectedness, and Mental Well-Being among Adolescents. Pak. J. Psychol. Res..

[22] Watson J.C., Haktanir A. (2017). School Connectedness, Self-Esteem, and Adolescent Life Satisfaction. J. Prof. Couns. Pract. Theory Res..

[23] Dovi A., Lindwall J., Sato T., Brigden J., Phipps S. (2019). Perceived school connectedness as it relates to parent-reported behavior and adaptive skills in youth with recently diagnosed cancer. Child. Health Care.

[24] Pikulski P.J., Pella J.E., Casline E.P., Hale A.E., Drake K., Ginsburg G.S. (2020). School connectedness and child anxiety. J. Psychol. Couns. Sch..

[25] Cohen S., Wills T.A. (1985). Stress, social support, and the buffering hypothesis. Psychol. Bull..

[26] Battistich V., Schaps E., Wilson N. (2003). Effects of an Elementary School Intervention on Students’ “Connectedness” to School and Social Adjustment During Middle School. J. Prim. Prev..

[27] Yu C., Zhang W., Zeng Y., Ye T., Li Y., Wang S. (2011). Relationship between adolescents’ gratitude and problem behavior: The mediating role of school connectedness. Psychol. Dev. Educ..

[28] Leadbeater B.J., Sukhawathanakul P., Thompson K., Holfeld B. (2015). Parent, Child, and Teacher Reports of School Climate as Predictors of Peer Victimization, Internalizing and Externalizing in Elementary School. Sch. Ment. Health.

[29] Sampson R.J., Raudenbush S.W. (1999). Systematic Social Observation of Public Spaces: A New Look at Disorder in Urban Neighborhoods. Am. J. Sociol..

[30] Mason M.J., Light J.M., Mennis J., Rusby J., Westling E., Crewe S., Zaharakis N.M., Way T., Flay B. (2017). Neighborhood disorder, peer network health, and substance use among young urban adolescents. Drug Alcohol Depend..

[31] Wang X., Maguire-Jack K. (2018). Family and Environmental Influences on Child Behavioral Health: The Role of Neighborhood Disorder and Adverse Childhood Experiences. J. Dev. Behav. Pediatr..

[32] Aneshensel C.S. (2009). Advances in the Conceptualization of the Stress Process.

[33] Ramey D.M., Harrington N. (2019). Early exposure to neighborhood crime and child internalizing and externalizing behaviors. Health Place.

[34] Coll C.G., Crnic K., Lamberty G., Wasik B.H., Jenkins R., García H.V., McAdoo H.P. (1996). An Integrative Model for the Study of Developmental Competencies in Minority Children. Child Dev..

[35] Roche K.M., Leventhal T. (2009). Beyond neighborhood poverty: Family management, neighborhood disorder, and adolescents’ early sexual onset. J. Fam. Psychol..

[36] Elkins I.J., McGue M., Iacono W.G. (1997). Genetic and environmental influences on parent-son relationships: Evidence for increasing genetic influence during adolescence. Dev. Psychol..

[37] Brown T.A. (2015). Confirmatory Factor Analysis for Applied Research.

[38] Harrington D. (2009). Confirmatory Factor Analysis.

[39] Resnick M.D., Bearman P.S., Blum R.W., Bauman K.E., Harris K.M., Jones J., Tabor J., Beuhring T., Sieving R.E., Shew M. (1997). Protecting adolescents from harm. Findings from the National Longitudinal Study on Adolescent Health. JAMA.

[40] He G.-H., Strodl E., Chen W.-Q., Liu F., Hayixibayi A., Hou X.-Y. (2019). Interpersonal Conflict, School Connectedness and Depressive Symptoms in Chinese Adolescents: Moderation Effect of Gender and Grade Level. Int. J. Environ. Res. Public Health.

[41] Ross C.E., Mirowsky J. (1999). Disorder and Decay: The concept and measurement of perceived neighborhood disorder. Urban Aff. Rev..

[42] Chang C.-W., Yuan R., Chen J.-K. (2018). Social support and depression among Chinese adolescents: The mediating roles of self-esteem and self-efficacy. Child. Youth Serv. Rev..

[43] Chen J.-K. (2020). Cyber victimisation, social support, and psychological distress among junior high school students in Taiwan and Mainland China. Asia Pac. J. Soc. Work Dev..

[44] Schafer J.L. (1997). Analysis of Incomplete Multivariate Data.

[45] Hayes A.F. (2017). Introduction to Mediation, Moderation, and Conditional Process Analysis: A Regression-Based Approach.

[46] Lerner R.M., Lerner J.V., von Eye A., Bowers E.P., Lewin-Bizan S. (2011). Individual and contextual bases of thriving in adolescence: A view of the issues. J. Adolesc..

[47] Sampson R.J., Raudenbush S.W., Earls F. (1997). Neighborhoods and Violent Crime: A Multilevel Study of Collective Efficacy. Science.

[48] Maguire-Jack K., Font S.A. (2017). Community and Individual Risk Factors for Physical Child Abuse and Child Neglect: Variations by Poverty Status. Child Maltreatment.

[49] Yoon S., Yoon D., Wang X., Tebben E., Lee G., Pei F. (2017). Co-development of internalizing and externalizing behavior problems during early childhood among child welfare-involved children. Child. Youth Serv. Rev..

[50] Wang W. (2013). Social workers involved in adolescent parent-child relationship conflict. Youth Soc..

[51] Liu Y., Carney J.V., Kim H., Hazler R.J., Guo X. (2019). Victimization and students’ psychological well-being: The mediating roles of hope and school connectedness. Child. Youth Serv. Rev..

[52] Forster M., Grigsby T.J., Gower A.L., Mehus C.J., McMorris B.J. (2020). The Role of Social Support in the Association between Childhood Adversity and Adolescent Self-injury and Suicide: Findings from a Statewide Sample of High School Students. J. Youth Adolesc..

[53] Sandel M., Faugno E., Mingo A., Cannon J., Byrd K., Garcia D.A., Collier S., McClure E., Jarrett R.B. (2016). Neighborhood-Level Interventions to Improve Childhood Opportunity and Lift Children Out of Poverty. Acad. Pediatr..

[54] Ibrahim S., Komulainen S. (2016). Physical punishment in Ghana and Finland: Criminological, sociocultural, human rights and child protection implications. Int. J. Hum. Rights Const. Stud..

